# FORWARD Study of GORE VIABAHN Balloon-Expandable Endoprostheses and Bare Metal Stents in the United States, European Union, United Kingdom, Australia, and New Zealand When Placed to Treat Complex Iliac Occlusive Disease: Protocol for a Randomized Superiority Trial

**DOI:** 10.2196/51480

**Published:** 2023-12-04

**Authors:** Melissa L Kirkwood, Ehrin J Armstrong, Mohammad M Ansari, Andrew Holden, Michel M P J Reijnen, Markus Steinbauer, Zachary Crannell, Hector Novoa, Austin Phillips, Darren B Schneider

**Affiliations:** 1 Division of Vascular and Endovascular Surgery The University of Texas Southwestern Medical Center Dallas, TX United States; 2 Advanced Heart and Vein Center Denver, CO United States; 3 Texas Tech University Health Science Center Lubbock, TX United States; 4 Vascular Intervention Research Unit Auckland City Hospital Auckland New Zealand; 5 Department of Surgery Rijnstate Arnhem Netherlands; 6 Multi-Modality Medical Imaging Group University of Twente Enschede Netherlands; 7 Vascular and Endovascular Surgery University of Regensburg Regensburg Germany; 8 W L Gore & Associates, Inc Flagstaff, AZ United States; 9 Division of Vascular Surgery and Endovascular Therapy University of Pennsylvania Philadelphia, PA United States

**Keywords:** iliac artery occlusive disease, VIABAHN VBX balloon expandable endoprosthesis, covered stent, stent graft, stent, randomized control trial, FORWARD, endoprosthesis, atherosclerosis, endovascular, stenting, occlusion, RCT, iliac occlusion

## Abstract

**Background:**

The recommendations for the use of and selection of covered stent grafts in patients with aortoiliac occlusive disease are limited.

**Objective:**

The GORE VBX FORWARD clinical study aims to demonstrate the superiority of the GORE VIABAHN VBX Balloon Expandable Endoprosthesis (VBX device) for primary patency when compared to bare metal stenting (BMS) for the treatment of complex iliac artery occlusive disease.

**Methods:**

A prospective, multicenter, randomized control study in the United States, European Union, United Kingdom, Australia, and New Zealand will enroll patients with symptomatic, complex iliac artery occlusive disease. In this study, iliac artery occlusive disease is defined as a unilateral or bilateral disease with single or multiple lesions (with >50% stenosis or chronic total occlusion) each between 4 and 11 cm in length. In an attempt to more closely match real-world practices, patients with minor tissue loss (Rutherford class 5) and patients requiring hemodialysis will be included. Baseline aortoiliac angiography will be performed to assess target lesion characteristics and determine final patient eligibility. Once the patient is confirmed and guidewires are in place across the target lesions, the patient will be randomized in a 1:1 format to treatment with either the VBX device or a BMS. The BMS can be balloon- or self-expanding and must be approved for the iliac artery occlusive disease indication. Patients, the independent core laboratory reviewers, and Clinical Events Committee members will be blinded from the assigned treatment. Dual antithrombotic medical therapy is required through a minimum of 3 months post procedure. The primary end point is 12‑month primary patency and will be adjudicated by an independent imaging core laboratory and Clinical Events Committee. Key secondary end points will be tested for superiority and include technical, acute procedural, and clinical success; changes in Ankle-brachial index; patient quality of life; primary patency; freedom from restenosis; primary-assisted patency; secondary patency; freedom from target lesion revascularizations; cumulative reintervention rate; amputation-free survival; survival; and change in Rutherford category. Study follow-up will continue through 5 years.

**Results:**

Outcomes will be reported following study completion. Enrollment is anticipated to start in October 2023.

**Conclusions:**

The results of this study will provide definitive, level 1 clinical evidence to clinicians on the optimal choice of stent device to use for the treatment of complex iliac artery occlusive disease. The FORWARD study is powered for superiority and includes only complex, unilateral, or bilateral lesions involving the common or external iliac arteries. This study is a multidisciplinary endeavor involving vascular surgery, interventional cardiology, and interventional radiology across multiple countries with a blinded core laboratory review of end points in hopes that the outcomes will be widely accepted and incorporated into practice guidelines for optimal treatment of patients with complex iliac artery occlusive disease.

**Trial Registration:**

ClinicalTrials.gov NCT05811364; https://clinicaltrials.gov/study/NCT05811364

**International Registered Report Identifier (IRRID):**

PRR1-10.2196/51480

## Introduction

Peripheral artery disease (PAD) of the lower extremities affects over 230 million adults worldwide and is the third leading cause of atherosclerotic morbidity behind coronary artery disease and stroke [1]. Symptomatic PAD, including lifestyle limiting claudication, rest pain, and tissue loss, is associated with significant disability, morbidity, and mortality. In the United States, the prevalence of PAD is estimated to be 7% to 10% [2,3], with approximately one-third involving the aortoiliac segment [4].

Endovascular treatment of aortoiliac occlusive disease (AIOD) has been proven to be a safe, durable, and efficient option compared to open repair, independent of the lesion Trans-Atlantic Inter-Society Consensus (TASC) II classification [5,6]. While balloon-expandable bare metal stents (BMS) have demonstrated acceptable technical success and 1-year primary patency in this location, balloon-expandable covered stents have arguably demonstrated more favorable primary patency through 12 months specifically in patients with complex TASC II C and D lesions [7,8]. Meta-analyses comparing BMS to balloon expandable covered stents suggest that covered stents may offer improved primary patency and freedom from reintervention, especially in more complex AIOD [9-11]. Randomized, controlled studies comparing balloon expandable covered stents and BMS in advanced AIOD, such as COBEST [12] and DISCOVER [13,14], have been conducted; however, limitations in these studies have prevented clear and conclusive outcomes. Despite only demonstrating a significant difference in binary restenosis in a subgroup analysis, the COBEST trial is widely referenced as the key evidence that supports the use of covered stents in advanced AIOD. While the COBEST trial subgroup analysis suggested a benefit to covered stenting, it should be considered hypothesis-generating for a future covered versus BMS comparative trial in complex iliac occlusive disease. The key limitations included a noninferiority design with minimal statistical power, limb-level randomization despite enrollment by TASC II classification, no independent core laboratory adjudication of end points, limited experience to 1 country (Australia), and limited follow-up to 18 months [12]. These limitations may contribute to the lack of additional recommendations for the use of covered stents in society consensus guidelines [15-17].

Though the Society of Vascular Surgery has published recommendations for the use of covered stent grafts in instances of severe calcification at risk of vessel rupture [15], there are no additional published guidelines on stent selection for AIOD [15,16,18]. The European Society for Vascular Surgery guidelines do recommend endovascular first treatment for patients with AIOD with severe comorbidities or in experienced teams if it does not compromise subsequent surgical options [16]. More recently, the DISCOVER trial compared the Advanta V12 (iCAST) to balloon-expandable BMS in “advanced” atherosclerotic disease of the common iliac artery [14]. This trial shares similar limitations to the COBEST trial [12] and focuses narrowly on the occlusive disease of the common iliac artery with a large proportion of TASCII A and B lesions (128/174, 73.5%) [14]. As a result, the DISCOVER trial failed to demonstrate any significant difference in primary or secondary outcomes at 2-year follow-up [14]. Other than the 5-year COBEST data [12], the literature for long-term, randomized comparison studies to serve as a guide for clinical practice for the treatment of AIOD remains limited.

The GORE VIABAHN VBX Balloon Expandable Endoprosthesis (VBX device) is a covered stent that has demonstrated favorable performance in patients with complex iliac occlusive disease. The FLEX Trial was a single-arm, nonrandomized study that reported a 1-year primary patency of 94.5%, with the overall cohort consisting of patients with kissing stents (57/134, 43%) and TASC C and D lesions (43/134, 32%) [8]. The GORE-sponsored FORWARD trial (ClinicalTrials.gov NCT05811364) is being conducted to demonstrate the superiority of treatment using the VBX device in patients with complex iliac artery occlusive disease when compared to BMS.

## Methods

### Study Design

The GORE VBX FORWARD clinical study is a prospective, multicenter, double-blind (patient, outcome assessor), randomized control trial comparing the VBX device to commercially available BMS in patients with complex iliac occlusive disease. The study is designed to randomize 244 patients at up to 40 sites across the United States, European Union, United Kingdom, Australia, and New Zealand.

### Ethical Considerations

**Figure 1:**
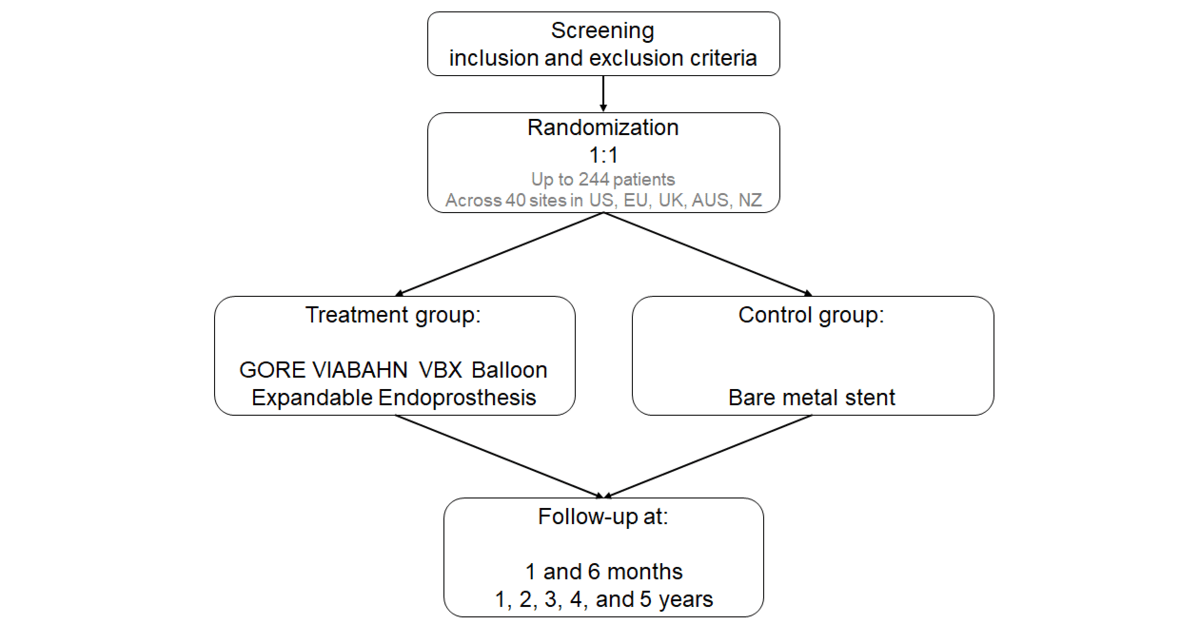
Study design schema. AUS: Australia; EU: European Union; NZ: New Zealand; UK: United Kingdom; US: United States.

The study protocol will be reviewed and approved by the institutional review board or ethics committee at each of the study sites. Patients will provide written informed consent before undergoing any study procedures and will be considered enrolled. The study does not involve the collection of identifiable patient information. Human participants enrolled in this study will be compensated in the amount of US $75 for returns to the 1-month, 6-month, 2-, 3- and 4-year follow-ups and US $100 for returns to the 1-year and 5-year follow-ups. The study will be conducted according to Good Clinical Practice, the Declaration of Helsinki guidelines, and in compliance with ISO 14155 (International Organization for Standardization). The study (design illustrated in Figure 1) is registered with ClinicalTrials.gov (NCT05811364).

### Study Population

The inclusion and exclusion criteria for the study are detailed in Textbox 1. Patients eligible for enrollment will be 18 years or older and have a symptomatic complex iliac occlusive disease with de novo or restenotic lesions in the common or external iliac arteries and with unilateral or bilateral, single or multiple lesions (>50% stenosis or chronic total occlusion) each between 4.0 and 11.0 cm in length (Textbox 1). In addition, clarifications to the specific lesion are (1) bilateral common iliac artery lesions that require kissing stents with at least 1 lesion >4 cm, (2) unilateral common iliac artery lesions that extend into the external iliac artery with a total lesion length >4 cm, (3) unilateral common iliac artery lesion >4 cm that are fully occluded somewhere in the lesion, and (4) multiple unilateral discrete (noncontiguous) lesions each >4 cm.

Preoperative and intraoperative inclusion criteria include symptomatic claudication, rest pain, or minor tissue loss (Rutherford category 2 to 5); reference vessel diameters ranging from 5.0 to 13.0 mm; a sufficient (<50% stenotic) common femoral; a sufficient (<50% stenotic) deep or superficial femoral artery; and a minimum of 1 sufficient (<50% stenotic) infrapopliteal run-off vessel (Textbox 1).

Exclusionary criteria include life expectancy <1 year; pregnancy; known allergy to stent or stent-graft components; nondialyzing patients with severe chronic renal insufficiency; evidence of a systemic infection; known intolerance to antithrombotic medications, vascular surgery, or catheterization of the lower extremities within 30 days of randomization; previous stenting in the iliac arteries; surgical bypass in the target limb; and current participation in another investigative clinical study. Intraoperative exclusions include a lesion requiring adjunctive procedures to facilitate stent delivery (eg, atherectomy and lithotripsy); abdominal aortic artery lesion or aneurysm; lesion requiring stent placement within 2 cm of inguinal ligament; and outflow disease requiring concomitant interventions. In addition, exclusionary are patients with isolated common iliac artery stenosis of any length that can be treated with a single device. For example, common iliac artery stenosis that does not require kissing stents or extending into the external iliac artery (Textbox 1) as these lesions are considered TASC A or B and would not be considered complex. While patients with bilateral disease are not excluded, those requiring concomitant procedures are excluded.

**Study inclusion and exclusion criteria.**

**Inclusion criteria**

- The study population has been defined as adult patients presenting with symptomatic, complex iliac artery occlusive disease. Patients must present with de novo or restenotic lesions (post percutaneous transluminal angioplasty) in the common or external iliac arteries, and complex iliac artery occlusive disease defined as unilateral or bilateral single or multiple lesions (>50% stenosis or chronic total occlusion) each between 4 and 11 cm in length.

Preoperative:

- Aged 18 years or older at the time of informed consent signature
- Informed Consent Form is signed by the patient
- The patient can comply with protocol requirements, including follow-up
- The patient has symptomatic claudication, rest pain, or minor tissue loss (Rutherford category 2-5)

Intraoperative:

- The patient has de novo or restenotic lesions found in the common or external iliac arteries
- The patient has: Unilateral or bilateral single or multiple lesions (>50% stenosis or chronic total occlusion) each between 4 and 11 cm in length
- The patient has a target vessel diameter visually estimated to be approximately between 5 and 13 mm
- The patient has a patent (<50% stenosis) common femoral artery and at least 1 patent (<50% stenosis) femoral artery (deep or superficial)
- The patient has at least 1 sufficient (<50% stenotic) infrapopliteal run-off vessel

**Exclusion criteria**

Preoperative:

- Life expectancy <1 year
- The patient is pregnant at the time of informed consent
- The patient has a known allergy to stent or stent graft components (including nitinol, stainless steel, or heparin)
- The patient has severe chronic renal insufficiency (serum creatinine level >2.5 mg/dL) and not undergoing hemodialysis
- The patient has evidence of a systemic infection
- The patient has a known intolerance to antithrombotic medications that prevent compliance with study or control device instructions for use
- The patient has had vascular catheterization of the lower extremities within 30 days of randomization (excluding diagnostic angiograms for the study procedure)
- The patient has previous stenting in the iliac arteries
- The patient has a previous surgical bypass in the target limb
- The patient is currently participating in another investigative clinical study unless received written approval from the sponsor

Intraoperative:

- The patient has a lesion requiring drug-coated balloon angioplasty, atherectomy, lithotripsy, or any ablative device to facilitate stent delivery
- The patient has an abdominal aortic artery lesion or aneurysm
- The patient has a lesion that requires stent placement within 2 cm of the inguinal ligament
- The patient has isolated common iliac artery stenosis that can be treated with a single device (ie, common iliac artery stenosis that does not require kissing stents or extend into the external iliac artery)
- The patient has outflow disease that requires concomitant interventions (ie, common femoral endarterectomy or femoral/tibial revascularization)

### Study Devices

The VBX device (W. L. Gore & Associates, Inc, Flagstaff, AZ; hereafter, VBX device) is comprised of a balloon-expandable stent graft with a balloon catheter delivery system. The VBX device is made of unconnected stainless-steel rings that are connected and enveloped by expanded polytetrafluoroethylene and fluorinated ethylene propylene film. The comparator device for the study is intended to be any commercially available BMS and can be balloon-expandable or self-expanding and must be approved for iliac artery occlusive disease indication. The use of interwoven stents or drug-eluting stents is not permitted in this study.

### Study Procedures

Patient screening and preprocedural imaging (eg, duplex ultrasound, computed tomography angiography, and diagnostic angiogram) will be performed at the discretion of the investigator and per the institution’s standard of care. Study measurements of medical history and demographics, laboratory workup, medication review, and physical examination will be collected. Vascular access, anticoagulation, and introduction of guidewires or catheters will be performed using the standard of care for iliac endovascular procedures at each institution. Baseline aortoiliac angiography will be performed to assess target lesion characteristics and determine final patient eligibility.

### Randomization and Blinding

Once study eligibility is confirmed and guidewires are in place across the target lesions, the patient will be randomized (1:1 format) to treatment with either the VBX device or a BMS (balloon-expandable or self-expanding and approved iliac artery occlusive disease indication). Permuted block randomization will be used to promote balance on baseline characteristics across treatment groups. The investigator at each site will be required to select devices that are consistent with the assigned treatment group and proceed with device implantation following the manufacturer’s instructions for use. Every effort will be made to limit the number of patient cross-overs (assigned treatment not performed).

Patients, the independent core lab reviewers, and Clinical Events Committee (CEC) members will be blinded from the assigned treatment. Given the nature of the procedure, the implanting investigator will not be blinded.

### Index Procedure and Postprocedure

All device implantations will be performed in compliance with the instructions for use for vessel preparation, sizing, delivery, positioning, and deployment. Predilation of each target lesion may be performed with plain balloon angioplasty. After the deployment of the devices, a postdilation may be performed, and a final angiography will be obtained to assess the technical result. Use of closure devices or sheath removal performed in accordance with institutional standard of care is at the investigator’s discretion. The procedure time (first incision to access site closure), anesthesia time, reference air kerma, and total fluoroscopic time will be recorded.

Secondary interventions to treat procedural complications requiring intervention will be performed at the discretion of the investigator and these postrandomized patients will continue to be followed at the protocol-specified intervals until study completion. Information about the angiographic images performed, devices and components used, the date of, and the adverse event leading to reintervention will be captured as part of the study record.

### Discharge and Follow-Up

At discharge, dual antithrombotic medical therapy is required through a minimum of 3 months post procedure. Additional single antiplatelet therapy (aspirin, clopidogrel, or prasugrel) is required for patients currently prescribed anticoagulant therapy (oral anticoagulation or direct oral anticoagulant). After at least 3 months, antithrombotic medical therapy should be continued post procedure per the investigator’s discretion. Patients will be evaluated by duplex ultrasound or angiography (if necessary) for patency at 1-month, 6-months, and annually through 5 years post randomization. Additional study assessments of ankle-brachial index (ABI) [19], Rutherford Classification [20], EQ-5D-5L Questionnaire [21], and the walking impairment questionnaire [22] will be captured.

### End Points

The primary end point is 1-year primary patency and is defined as blood flow through the target lesion (no evidence of binary restenosis >50% or occlusion) without target lesion revascularization (TLR). Primary patency will be evaluated and adjudicated by the independent vascular Core laboratory. TLR events will be reviewed and adjudicated by the independent CEC (Table 1).

Secondary end points (defined in Table 1) will include technical success, acute procedural success, clinical success (through 30 days), changes in ABI (hemodynamic status), EQ-5D-5L, and walking impairment questionnaire through 5 years, and the following through 5 years: primary patency, freedom from binary restenosis, primary-assisted patency, secondary patency, freedom from TLR, cumulative reintervention rate (first and recurrent TLR), freedom from clinically-driven TLR, amputation-free survival, survival, and change in Rutherford category.

**Table 1:** Study end point definitions.

Term	Definition
Primary patency	Blood flow through the target lesion (no evidence of binary restenosis >50% or occlusion) without a TLR^a^
Binary restenosis	Evidence of >50% restenosis or occlusion of the target lesions based on core laboratory adjudicated duplex ultrasound or angiography
Primary assisted patency	Blood flow maintained (no evidence of occlusion) through the target lesion with or without a TLR
Secondary patency	Blood flow through the target lesion with or without a TLR
Target lesion revascularization	Endovascular or surgical intervention performed on the target lesions
Clinically driven target lesion revascularization	Endovascular or surgical intervention performed on the target lesions in response to recurrent symptoms (increase ≥1 Rutherford category)
Amputation-free survival	Freedom from major amputation (target limb, amputation above the metatarsals) and all-cause mortality
Survival	Freedom from all-cause mortality
Technical success	Deployment of device with <30% residual stenosis on final angiography
Acute procedural success	Technical success and freedom from device or procedure-related SAE^b^ requiring intervention
Clinical success	Improvement from baseline of ≥1 Rutherford category and freedom from device or procedure-related SAE requiring intervention
Hemodynamic status	Change in ABI^c^ as compared to baseline
Rutherford category status	Change in Rutherford category as compared to baseline
Quality of life assessments	Change in EQ-5D-5L responses as compared to baseline Change in WIQ^d^ responses as compared to baseline

^a^TLR: target lesion revascularization.

^b^SAE: serious adverse event.

^c^ABI: ankle-brachial index.

^d^WIQ: walking impairment questionnaire.

### Statistical Analysis

This study is designed to test the null hypothesis that the VBX device group will demonstrate a primary patency that is less than or equal to the BMS group at 1-year follow-up; versus the alternative hypothesis that the VBX device group will demonstrate a primary patency that is greater than the BMS at 1-year follow-up.

### Sample Size Considerations

This study is designed with the assumption that 1-year primary patency will be 88% and 70% for the VBX device group and BMS group, respectively. The study assumptions are based on the FLEX clinical study data and a recent meta-analysis of covered stents and BMS (both balloon-expandable and self-expanding) in patients with TASC II C and D lesions with a minimum of 12-month follow-up [11].

A total sample size of 244 patients would be required to account for a 20% loss to follow-up in order to demonstrate statistical significance (α=.05) at a power of 88%.

### Primary and Secondary End Point

The study analysis plan will include the following analysis populations: (1) intent-to-treat population (randomized patients categorized by assigned treatment); (2) as-treated population (randomized patients categorized by treatment received); and (3) per-protocol population. Primary patency at 1 year post implantation of the VBX device and BMS treatment groups will be analyzed with Kaplan-Meier estimates with separate estimates for each group. A 2-sided confidence interval of these estimates (α=.05) will be constructed to test the observed differences in primary patency rates between the treatment groups.

A comparative analysis of secondary end points will be performed. If the null hypothesis for the primary end point is rejected, key secondary end points will be evaluated for superiority using fixed sequential testing. In order, the secondary end points to be tested for superiority are cumulative target revascularization rate (first and recurrent events), the difference in mean ABI, and freedom from TLR.

Time-to-event secondary end points will be analyzed with Kaplan-Meier estimates with separate estimates for each group. Categorical end points for each treatment group will be analyzed with chi-square tests, and continuous end points for the treatment groups will be analyzed with 2-sample *t* tests.

### Trial Organization and Oversight

The Executive Steering Committee, consisting of multidisciplinary (vascular surgery, interventional cardiology, and interventional radiology) key opinion leaders, will provide study guidance and clinical oversight. The sponsor will provide study oversight in partnership with contract research organizations for database development, data monitoring, and independent review of study outcomes.

All imaging required by the study including duplex ultrasound and angiography, will be sent to the independent study core laboratory for centralized review and adjudication. An independent CEC will review and adjudicate potential end point–related events. For end point analysis, the sponsor will use data adjudicated by the core laboratory and CEC.

## Results

Site enrollment is currently in process, and participant enrollment is anticipated to begin in October of 2023. Participant enrollment is expected to take up to 32 months. Once all participants are enrolled, follow-up will continue through 5 years.

## Discussion

### Progressive Standards

The literature for long-term, randomized comparison studies for the treatment of AIOD is limited and, therefore, there are no accepted, published guidelines regarding the selection of either bare metal or covered stents. The COBEST trial [12] is the reference data often cited supporting the use of covered stents in complex AIOD. However, the trial was a noninferiority study with minimal statistical power, limited experience in 1 country, only an 18-month follow-up visit, and no independent core laboratory review for adjudication of end points. Furthermore, the COBEST trial was designed to evaluate binary restenosis rather than primary patency. The DISCOVER trial [13,14] failed to show any benefit of covered stents in “advanced” common iliac artery disease across all end points, likely due to the majority of TASC II A and B lesions. The FLEX trial included patients with tortuous iliac arteries, severe calcification, chronic total occlusions, and those who required kissing stents at the aortic bifurcation [7]. Importantly, despite the inclusion of more challenging iliac pathology, the FLEX study demonstrated a 1-year patency of 94.5% for the VBX stent-grafts in the treatment of AIOD involving the common or external iliac arteries [8]. The physician-initiated long-term follow-up of the FLEX study [23], demonstrated a 5-year patency of 89.5% which appears significantly greater than the 5-year patency of 74.7% for covered stents in the COBEST study [12]. While these studies suggest that the primary patency of covered stents may be better than BMS for the treatment of complex AIOD, there is a lack of level 1 evidence and no clear consensus regarding the optimal stent selection in AIOD.

The objective of the FORWARD study is to demonstrate the superiority of the VBX device for primary patency when compared to BMS in complex iliac occlusive disease in a prospective, multicenter, randomized controlled trial. The data from this study aims to provide level 1 evidence that will significantly enhance therapeutic decision-making and help establish a standard of care to guide clinical practice for patients with symptomatic severe aortoiliac disease. The FORWARD study is powered for superiority and includes only complex, unilateral, or bilateral lesions involving the common or external iliac arteries. To mirror real-world practice more closely, patients with minor tissue loss (Rutherford class 5) and patients requiring hemodialysis will be included. This study is a multidisciplinary endeavor involving vascular surgery, interventional cardiology, and radiology across multiple countries with a blinded core laboratory review of end points in hopes that the outcomes will be widely accepted by all parties that treat symptomatic patients with complex AIOD.

### Limitations

In the design of this study, multiple considerations were taken to curb the number of limitations. While the study intends to target complex diseases, the results may not be generalizable to populations with less complex diseases. This population is less frequent and may slow enrollment. The study is powered to demonstrate superiority at 1 year, however, longer-term outcomes are also clinically relevant. Additionally, due to the nature of the procedure, blinding of the implanting investigator is not possible which has the potential to induce bias.

## Appendix

Multimedia Appendix 1 CONSORT 2010 Checklist.

## Abbreviations

ABI: ankle-brachial index
AIOD: aortoiliac occlusive disease
BMS: bare metal stents
CEC: Clinical Events Committee
ISO: International Organization for Standardization
PAD: peripheral artery disease
TASC: Trans-Atlantic Inter-Society Consensus
TLR: target lesion revascularization
VBX device: GORE VIABAHN VBX Balloon Expandable Endoprosthesis

## Data Availability

Data generated or analyzed during this study will be included in peer-reviewed publications. The data sets have yet to be generated or analyzed for this study because no participants have been enrolled.
